# Habitat-dependent composition of bacterial and fungal communities in biological soil crusts from Oman

**DOI:** 10.1038/s41598-019-42911-6

**Published:** 2019-04-23

**Authors:** Raeid M. M. Abed, Alexandra Tamm, Christiane Hassenrück, Ahmed N. Al-Rawahi, Emilio Rodríguez-Caballero, Sabine Fiedler, Stefanie Maier, Bettina Weber

**Affiliations:** 1 Sultan Qaboos University, College of Science, Biology Department, P.O. Box: 36, postal code 123 Al Khoud, Sultanate of Oman; 2 Multiphase Chemistry Department, Max Planck Institute for Chemistry, Hahn-Meitner-Weg 1, D-55128 Mainz, Germany; 3 Tropical Marine Microbiology, Department of Biogeochemistry and Geology, Leibniz Centre for Tropical Marine Research, Bremen, Germany; 4 Departamento de Agronomia, Universidad de Almeria, Almería, Spain; 5 Johannes Gutenberg-University, Institute for Geography, Mainz, Germany

**Keywords:** Soil microbiology, Microbial ecology

## Abstract

Biological soil crusts (biocrusts) occur within drylands throughout the world, covering ~12% of the global terrestrial soil surface. Their occurrence in the deserts of the Arabian Peninsula has rarely been reported and their spatial distribution, diversity, and microbial composition remained largely unexplored. We investigated biocrusts at six different locations in the coastal and central deserts of Oman. The biocrust types were characterized, and the bacterial and fungal community compositions of biocrusts and uncrusted soils were analysed by amplicon sequencing. The results were interpreted based on the environmental parameters of the different sites. Whereas at lowland sites, mainly cyanobacteria-dominated biocrusts were observed, both cyanobacteria- and lichen-dominated biocrusts occurred at mountain sites. The majority of bacterial sequences (32–83% of total sequences) belonged to Actinobacteria, Cyanobacteria, Alphaproteobacteria, and Bacteroidetes, whereas fungal sequences belonged to Ascomycota, Basidiomycota, and Chytridiomycota (>95%). With biocrust development, a notable increase in cyanobacterial and decrease in actinobacterial proportions was observed for cyanobacteria-dominated crusts. In coastal areas, where salinity is high, biocrusts were replaced by a unique marine mat-like microbial community, dominated by halotolerant taxa. Redundancy analysis revealed a significant contribution of soil texture, cover type, carbon content, and elevation to the variations in bacterial and fungal communities. Multivariate analysis placed microbial communities in significantly separated clusters based on their carbon content, elevation and electrical conductivity. We conclude that Oman hosts a variety of cyanobacteria- and lichen-dominated crusts with their bacterial and fungal communities being largely dictated by soil properties and environmental parameters.

## Introduction

Biological soil crusts (biocrusts), composed of cyanobacteria, algae, lichen and mosses, are distributed worldwide in diverse environments ranging from hot to cold deserts and are estimated to cover 30–40% of the total arid and semi-arid landscapes^1–4^. The distribution patterns and heterogeneity of biocrusts can differ depending on the spatial scale, and they are often dictated by several biotic (e.g. plant cover and human disturbance) and abiotic (e.g. climatic and edaphic) factors^5–12^. These parameters can be distinct, but can also interrelate and depend on each other, and some vary spatially (from meters to kilometers) and temporally (days or seasons). Several studies have been devoted to gain a better understanding of the different parameters that determine the heterogeneity of biocrusts and their microbial communities^10–15^. Biocrust communities varied among different geographical locations within Africa, Australia, Asia and North America^14,16–18^ and even when comparison was carried out across continents and between hot and temperate deserts^10,19,20^. Patchiness in biocrust microenvironments has even been detected at the microscale level, using microsensors, indicating that different microhabitats supported the growth of different microorganisms^2^. Early studies from the arid and semi-arid Australia revealed a significant relationship between crusts’ species composition and soil physical and chemical properties^11–14^. Climatic changes in temperature and precipitation lead to replacement of certain biocrust species by others^21,22^. Soil properties including texture class, pH, salinity, nutrients and porosity strongly correlated with the distribution patterns of biocrust species^23,24^. Anthropogenic disturbance has also been shown to reduce diversity, abundance and activity of biocrust microorganisms^25–28^.

Most of the research related to distribution patterns of biocrusts was performed on samples originating from Australia, North America and Africa, while there is a clear gap in our knowledge on the biogeography of biocrust microorganisms on the Arabian Peninsula. Moreover, only recently modern molecular techniques have been used to correlate species compositions of biocrusts to their environments^17,29^. On the Arabian Peninsula, deserts are a prominent feature, covering more than half of the total area and characterized by limited plant cover. Deserts in this region (e.g. Oman) vary from semiarid in the central desert to arid in the coastal regions, with clear differences in thermal regimes and precipitation rates. While the central desert experiences freezing temperatures and an annual rainfall of ca. 300 mm during November to March, these values may reach 40 °C and <100 mm in the coastal deserts. Some biocrusts in coastal regions are exposed to frequent inundation by seawater, resulting in a clear transition from completely desiccated biocrusts to salt-influenced biocrusts, which are then considered as intertidal marine microbial mats. So far, the distribution of biocrusts and the parameters that drive the heterogeneity of their microbial communities have not been investigated for Oman. Such information provides the baseline knowledge that can be used to predict vegetation development and consequences of climate change (e.g. soil warming and altered precipitation) in the future.

**Figure 1:**
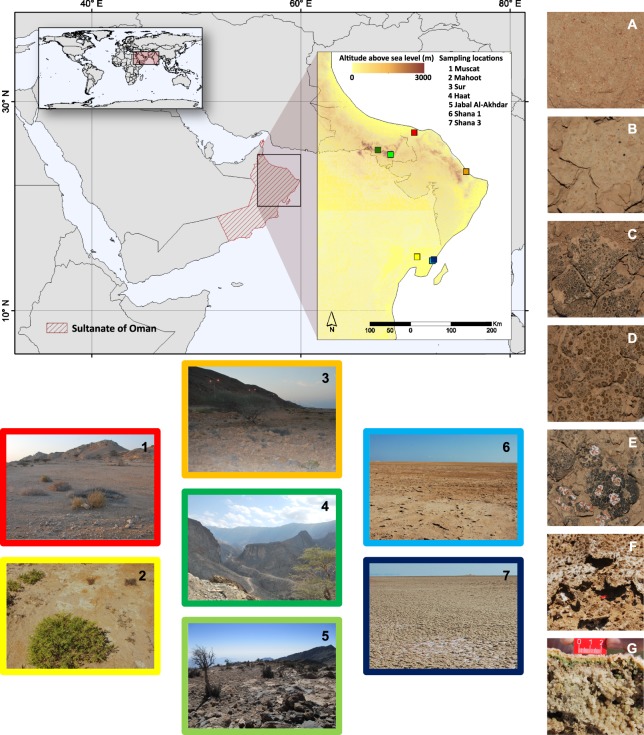
Map composition presenting the sampling locations and sample types in the Sultanate of Oman. Framed and serially numbered pictures show the different sampling locations. On the right hand side close-up pictures of different biocrust types and bare soil samples are shown (**A**) bare soil; (**B**) cyanobacteria-dominated biocrust; (**C**) cyanobacteria-dominated biocrust with cyanolichen; (**D**) chlorolichen-dominated biocrust with *Placidium* sp.; (**E**) chlorolichen-dominated biocrust with *Psora* sp. and cyanolichens; (**F**) salt crust with microbial communities growing inside the crust; (**G**) soft, marine microbial mats (cross section)). Topographic information obtained from the NASA Shuttle Radar Topography Mission (SRTM^30^). All maps were created using ArcGIS Desktop 10.3 (Copyright(©) 1995–2015 ESRI; License acquired by the university of Almeria thought the agreement between University and Centro Informático Científico de Andalucía, CICA). Country boundaries were obtained from the FGGD coastal and country boundaries of the world 1.0 (2007; http://ref.data.fao.org/map?entryId = 18329470-472d-11db-88e0-000d939bc5d8&tab = metadata).

In this study, we expand our understanding of the biogeography and species composition of different biocrust types from Oman and explore the determinants that contribute to the variation of their bacterial and fungal communities. We collected samples of bare soils, cyanobacteria- and lichen-dominated crusts from six localities (Fig. 1). Marine microbial mats from only one location (i.e. Shana) were included in our sampling for comparison of microbial communities with salt-influenced biocrusts. The sampling sites included the coastal and the central desert, covered an altitudinal gradient, and were chosen because of the clear variability in climatic and edaphic properties.

## Materials and Methods

### Study sites and sample collection

Sampling was carried out at six different locations (termed as Jabal Al-Akhdar, Muscat, Mahoot, Sur, Haat, and Shana) in the Northern and Eastern parts of the Sultanate of Oman in January 2016. The chosen sites represent variable environmental and ecological conditions, differing in soil texture, elevation, climatic conditions, human disturbance and distance to the sea (Table 1). A map depicting the sampling locations was created using ArcGIS Desktop 10.3 (Copyright(C) 1995–2015 ESRI). Topographic information were obtained from the NASA Shuttle Radar Topography Mission (SRTM^30^).

**Table 1:** Sample locations and soil characteristics of bare soils, biocrusts and mats from the Sultanate of Oman.

Sampling sites	Type/	Latitude	Longitude	Elevation (m)	Disturbance	pH	Conductivity	N content	C content	Soil texture	FAO*
	Replicate	(°)	(°)				(µS/m)	(%)	(%)	Major class	
Muscat	Bare1	23.580318	58.201758	38	+	8.8	114	0.00	9.73	Sand	S (sand)
	Bare2	23.580318	58.201758	38	+	8.6	210	0.01	10.00	Sand	S (sand)
	Bare3	23.580318	58.201758	38	+	8.7	189	0.01	8.49	Sand	S (sand)
	Bare4	23.580318	58.201758	38	+	8.8	120	0.01	7.20	Sand	S (sand)
	Cyano1	23.580318	58.201758	38	+	7.8	1930	0.08	6.01	Clay	SiC (silt clay)
	Cyano2	23.580318	58.201758	38	+	7.9	301	0.16	6.03	Loam	SCL (sandy clay loam)
	Cyano3	23.580318	58.201758	38	+	8.1	175	0.11	6.16	Loam	CL (clay loam)
Sur	Bare1	22.714222	59.353199	20	+	8.5	128	0.04	7.51	Sand	LS (loamy sand)
	Bare2	22.714222	59.353199	20	+	8.6	256	0.03	9.14	Sand	LS (loamy sand)
	Bare3	22.714222	59.353199	20	+	8.7	148	0.02	6.95	Sand	LS (loamy sand)
	Bare4	22.714222	59.353199	20	+	8.5	130	0.03	7.93	Sand	LS (loamy sand)
	Cyano1	22.714222	59.353199	20	+	8.6	148	0.04	6.37	Clay	SC (sandy clay)
	Cyano2	22.714222	59.353199	20	+	8.4	245	0.03	6.47	Clay	SiC (silt clay)
	Cyano3	22.714222	59.353199	20	+	8.7	107	0.03	6.41	Clay	SiC (silt clay)
Mahoot	Bare1	20.829529	58.266045	37	+	8.6	166	0.01	3.18	Loam	SL (sandy loam)
	Bare2	20.829529	58.266045	37	+	8.7	370	0.01	4.00	Loam	SL (sandy loam)
	Bare3	20.829529	58.266045	37	+	8.7	1228	0.02	3.41	Loam	SL (sandy loam)
	Bare4	20.829529	58.266045	37	+	8.6	1134	0.01	2.42	Loam	SL (sandy loam)
	Cyano1	20.829529	58.266045	37	+	8.3	228	0.03	3.65	Loam	SiL (silt loam)
	Cyano2	20.829529	58.266045	37	+	8.5	232	0.01	3.88	Loam	SCL (sandy clay loam)
	Cyano3	20.829529	58.266045	37	+	8.2	584	0.01	3.49	Loam	SCL (sandy clay loam)
Haat	Bare1	23.193069	57.396991	989	−	8.3	97	0.15	5.19	Sand	LS (loamy sand)
	Bare2	23.193069	57.396991	989	−	8.4	160	0.15	5.03	Sand	LS (loamy sand)
	Bare3	23.193069	57.396991	989	−	8.3	121	0.16	4.16	Sand	LS (loamy sand)
	Bare4	23.193069	57.396991	989	−	8.3	110	0.22	5.28	Sand	LS (loamy sand)
	Cyano1	23.193069	57.396991	989	−	8.2	160	0.14	6.25	Loam	SL (sandy loam)
	Cyano2	23.193069	57.396991	989	−	8.3	115	0.16	5.14	Loam	SL (sandy loam)
	Cyano3	23.193069	57.396991	989	−	8.4	91	0.18	4.56	Clay	SiC (silt clay)
	Lichen1	23.193069	57.396991	989	−	8.5	95	0.21	4.41	Sand	LS (loamy sand)
	Lichen2	23.193069	57.396991	989	−	8.1	171.2	0.14	7.33	Clay	SiC (silt clay)
	Lichen3	23.193069	57.396991	989	−	8.5	103	0.17	5.40	Clay	SiC (silt clay)
Jabal Al-Akhdar	Cyano1	23.091781	57.680719	2022	−	8.3	341	0.17	7.88	Loam	CL (clay loam)
	Cyano2	23.091781	57.680719	2022	−	8.4	111	0.08	4.91	Clay	SC (sandy clay)
	Cyano3	23.091781	57.680719	2022	−	8.3	122	0.12	5.27	Loam	SCL (sandy clay loam)
	Lichen1A	23.091781	57.680719	2022	−	8.2	184.3	0.13	5.91	Loam	SiL (silt loam)
	Lichen2A	23.091781	57.680719	2022	−	8.0	255	0.22	6.24	Loam	CL (clay loam)
	Lichen3A	23.091781	57.680719	2022	−	8.3	133	0.12	4.43	Clay	SiC (silt clay)
	lichen1B	23.091781	57.680719	2022	−	8.4	133	0.13	5.14	Clay	SiC (silt clay)
	Lichen2B	23.091781	57.680719	2022	−	8.3	100	0.12	5.39	Clay	SiC (silt clay)
	Lichen3B	23.091781	57.680719	2022	−	8.2	140	0.18	4.58	Loam	CL (clay loam)
Shana1	Cyano1	20.743597	58.603713	7	−	8.3	65800	0.01	2.30	Sand	S (sand)
	Cyano2	20.743597	58.603713	7	−	8.3	45900	0.00	2.40	Sand	S (sand)
	Cyano3	20.743597	58.603713	7	−	8.3	108800	0.01	1.93	Sand	S (sand)
Shana3	Mat1	20.769648	58.639686	12	−	8.9	34700	0.06	0.67	Sand	S (sand)
	Mat2	20.769648	58.639686	12	−	9.1	34800	0.05	0.60	Sand	S (sand)
	Mat3	20.769648	58.639686	12	−	9.5	35400	0.04	0.54	Sand	S (sand)

*According to FAO (2006): Guidelines for soil description http://www.fao.org/3/a-a0542e.pdf.

Haat and Jabal Al-Akhdar are both situated within the Al-Hajar Mountain ranges and sampling sites were located at an elevation of approx. 989 and 2022 m a.s.l., respectively. Haat and Jabal Al-Akhdar are fairly pristine locations, characterized by a stony and rocky ground with small areas or sinks in between, where biocrusts and bare soil can be found. All other samples were collected at lowland sites ranging between 7 to 38 m a.s.l. (Table 1). The locations Mahoot, Muscat and Sur are anthropogenically influenced areas. The Mahoot site, about 25 km away from the sea, was situated next to a big road, where tyre and hoof prints indicated anthropogenic influences as well as an impact by grazing. The sampling site Sur was located between a highway road and the sea, whereas the Muscat site was a ruderal site located at the edge of the city of Muscat. While the non-elevated sites experience temperatures between 40–50 °C in summer and as low as 12 °C in winter, with a mean annual precipitation of ca. <100 mm (from November to March), the temperature at the elevated sites reaches a maximum of 25–35 °C in summer but can freeze in winter, with a mean annual precipitation of about 300 mm^31^. The Shana samples were collected close to the sea under marine influence. Shana1 was a salt pan area presenting hard and desiccated salt crusts with interspersed microbial communities, whereas Shana3 was inundated by seawater during high tides, forming soft marine mats with a typical striped appearance.

At each site, samples of the different types of biocrusts as well as bare soils were collected in triplicates. The topmost 1 cm of cyanobacteria- and lichen-dominated crusts (when found) as well as bare soils (for comparison) were collected in small petri dishes (5.5 cm diameter) using aseptic tools. Biocrusts from Shana1 and microbial mats from Shana3 sites were also sampled in triplicate in a similar way. Samples for molecular work were transported on dry ice to guarantee a continuous cooling chain and finally stored in a freezer at −80 °C until analysis.

### Environmental and soil quality parameters

The textural classes were estimated by a simple field test based on feeling the constituents of the soil^32^. For this, soil samples have to be in a moist to slightly wet state and gravel and other constituents >2 mm have to be removed. The constituents feel as follows, depending on the dominating compound: (1) Clay, such as clayey loam, feels sticky. Soil between fingers, is cohesive (sticky), is formable, has a high plasticity and a shiny surface when squeezed between fingers; (2) Silt, such as silty loam or silty clay, feels smooth. Soil between fingers, is non-sticky, only weakly formable, has a rough and rippled surface after squeezing between fingers and feels very floury (like talcum powder), and (3) Sand, such as sandy loam or sandy clay, has a gritty texture. It cannot be formed, does not stick to fingers and feels very grainy.

For determination of soil pH and electrical conductivity, 1 g of soil were thoroughly mixed with 2.5 ml of distilled water and allowed to stand for 45 min. The filtrate was collected and the pH and electrical conductivity were measured from the filtrate using glass electrodes (Minitrode, Hamilton Messetechnik GmbH, Höchst, Germany). Total nitrogen and carbon were analysed in the soil samples using a C/N analyser (cube EL, Elementar Analysensysteme GmbH, Hanau, Germany), after air drying the soil, followed by grinding and homogenization. The results are expressed as oven-dry soil (105 °C).

### DNA sequencing

For DNA extraction, we combined many small subsamples from one petridish to obtain one DNA sample per petridish. The subsamples of the biocrusts and mats were taken from the photoautotrophic layer and the heterotrophic layer directly below. For bare soil, many subsamples taken from the uppermost millimetres were combined in one sample. DNA was extracted from biocrusts, bare soils and microbial mats (total 45 samples) using PowerSoil DNA extraction kit (MOBIO laboratories, Inc., Carlsbad, CA) according to the manufacturer’s instructions. Purified DNA extracts were then submitted to Molecular Research MR DNA laboratory (www.mrdnalab.com, Shallowater, TX, USA) for paired-end Illumina amplicon sequencing of the bacterial 16S rRNA genes V4 variable region using the primers 341F (5′-CCTACGGGNGGCWGCAG-3′) and 805R (5′-GACTACHVGGGTATCTAATCC-3′) with barcode on the forward primer^33^. A single-step 30 cycle PCR using HotStarTaq Plus Master Mix kit (Qiagen, USA) was performed at 94 °C for 30 seconds, 53 °C for 40 seconds and 72 °C for 1 minute. The fungal internal transcribed spacer (ITS) region was also amplified from the same DNA extracts using the primers ITS1F (5′-CTTGGTCATTTAGAGGAAGTAA-3′) and ITS2R (5′-GCTGCGTTCTTCATCGATGC-3′)^34^. After amplification, PCR products were checked in 2% agarose gel to determine the success of amplification and the relative intensity of bands. Multiple samples were pooled together in equal proportions based on their molecular weight and DNA concentrations. Pooled samples were purified using calibrated AMPure XP beads according to the manufacturer’s instructions (BioLabs, NewEngland). The pooled and purified PCR products were used to prepare a DNA library by following Illumina DNA library. Details for library preparation protocol and quality control can be found in TrueSeq Nano DNA Library Prep, Reference Guide, Illumina. Sequencing was performed on a MiSeq following the manufacturer’s guidelines.

Sequence data were demultiplexed and adapters and primers were removed using the software *FASTqProcessor* version 1.1.4.19846 (http://www.mrdnafreesoftware.com/; date accessed: 23.02.2019) provided by MR DNA (MR DNA, Shallowater, TX, USA). For the fungal data set, primers were further removed from the 3′ end of each read using *cutadapt* version 1.9.1^35^. In compliance with the Minimal Information about any (X) Sequence (MIxS) standard^36^, the demultiplexed and primer-clipped sequences were deposited at the European Nucleotide Archive^37^ using the data brokerage service of the German Federation for Biological Data^38^. They are accessible under the project accession number PRJEB31699. Further sequence processing steps were conducted in R version 3.5.2^39^ using the package *dada2* version 1.10.1^40^. Briefly, sequences were quality filtered at a maximum expected error rate of 3 after trimming both forward and reverse reads to 230 bp (bacterial data set) or removing reads shorter than 50 bp (fungal data set). Error learning, dereplication, and denoising were conducted with default parameters. Forward and reverse reads were merged with a minimum overlap of 10 bp. For chimera detection and taxonomic classification default settings were selected. Bacterial sequences were classified using the SILVA reference database version 132^41^ and fungal sequences were classified using the fungal release of the UNITE reference database^42^. Only non-singleton and non-doubleton sequence variants, hereafter referred to as operational taxonomic units (OTUs), that were classified on phylum level were retained for further analysis. Chloroplast and mitochondrial sequences were further removed from the bacterial data set. The code for the sequence analysis has been submitted to PANGAEA alongside the environmental and soil parameters (10.1594/PANGAEA.899529).

### Statistical analysis

A principle component analysis (PCA) was conducted to discern patterns among samples based on their environmental characteristics. The following parameters were included in the PCA calculation using the R package PCAmixdata (version 3.1)^43^: elevation, pH, electrical conductivity, carbon and nitrogen content, human disturbance, soil texture class, and soil cover type (i.e. bare, cyano and lichen). For simplicity, textural classes were divided into sand, loam, and clay according to the dominant particle size.

Alpha diversity of the bacterial and fungal communities was assessed based on richness (number of OTUs), the Shannon-Wiener Index, and the Inverse Simpson Index after randomly rarefying the data set repeatedly to the minimum library size (bacteria: 16,338 sequences; fungi: 15,827 sequences). Samples with less than 10,000 sequences were excluded from the alpha diversity analysis. Cluster analysis and non-metric multidimensional scaling (NMDS) were performed to visualize patterns in bacterial and fungal community composition. While for the cluster analysis Bray-Curtis dissimilarities were calculated based on relative sequence abundances of OTUs without further transformation, for NMDS, the Wisconsin double standardization was applied to the relative sequence abundances prior to the calculation of Bray Curtis dissimilarities. Bacterial communities collected at Shana were excluded from the NMDS since their Bray-Curtis dissimilarity to any of the other samples was by far the highest between any two samples, which would have reduced the resolution of the dissimilarities between the remaining samples. Analysis of similarity (ANOSIM) was used to assess the separation of bacterial and fungal communities between groups of samples based on similar environmental parameters.

Redundancy analyses (RDA) were run to evaluate the ability of environmental parameters (cover type, soil texture, human disturbance, elevation, electrical conductivity, pH, carbon and nitrogen content) to explain the variation in bacterial and fungal community composition. Nitrogen content was excluded as an explanatory variable in the RDA models due to a high collinearity with elevation. Prior to RDA, OTUs that did not occur in at least 2 replicates per location and sample type were removed from the dataset. Furthermore, sequence counts were centered log ratio (clr)-transformed. Best-fitting RDA models were selected based on maximum adjusted R² and minimum AIC (Akaike Information Criterion, an estimator of the relative quality of statistical models for a given dataset). Variance inflation factors of the explanatory variables in the best-fitting models were below 10. All statistical analyses were conducted using the R software package, version 3.5.1^39^ and R-Studio, version 1.0.153^44^. Additional packages used within R were ‘PCAmixdata’^43^ and ‘vegan’^45^.

## Results

### Variation among sampling sites and soil samples

**Figure 2:**
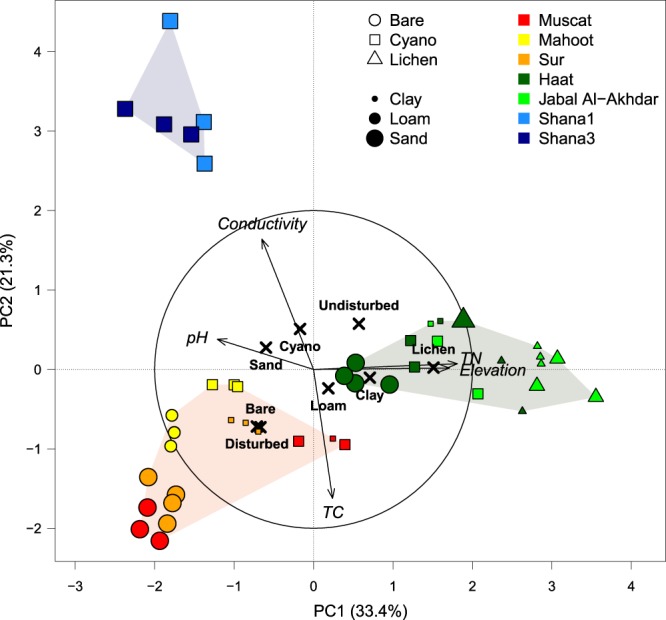
PCA plot of environmental parameters. Color, shape, and size of points indicate sampling location, surface cover type, and soil texture, respectively. Based on their environmental characteristics, samples group into 3 distinct categories, shown as shaded hulls: disturbed (red), marine (blue), and elevated (green) sites. TC and TN refer to total carbon and total nitrogen, respectively.

Field observations showed that cyanobacteria-dominated crusts were encountered at all sampling sites, whereas lichen-dominated crusts were mainly restricted to the elevated sites (i.e. Haat and Jabal Al-Akhdar). Bare soils (except from Mahoot) and Shana biocrusts and mats had coarse sandy texture, all Mahoot soils had loamy texture and the remaining biocrusts had either loamy or clayey texture (Table 1). A PCA analysis captured 54.7% of the variation among sampling sites on the first two principle components using the measured environmental parameters and soil characteristics (Fig. 2). Elevation of the sampling site co-varied with nitrogen (N) content in soils and both were the major contributors to PC1, which accounted for 33.4% of the variation. PC2 explained 21.3% of total variance and this was mainly attributed to electrical conductivity and carbon content. Based on the PCA patterns, samples collected from elevated sites (i.e. Jabal Al-Akhdar and Haat) were clearly separated from the rest (Green hull in Fig. 2). These soils had the highest N levels with values between 0.08 and 0.22% (Table 1). The sampling sites Mahoot, Sur and Muscat, characterized by their exposure to human and/or animal disturbance, clustered together (Red hull in Fig. 2). Shana biocrust and mat samples formed a distinct cluster along the second PC axis, significantly different from all other samples (Blue hull Fig. 2). This separation was largely driven by the high electrical conductivity values, which reached as high as ca. 109 mS/m (Table 1).

Redundancy analysis revealed that soil cover type, soil texture, carbon content, elevation, and human disturbance explained 32% of the variation in bacterial communities but only 19% in fungal communities (Table 2). Individually, all tested parameters contributed significantly to the variations in bacterial communities while accounting for the effect of the other parameters (pure effects), although the total effects were often considerably larger. Among the observed environmental parameters, the most important determinants of bacterial communities were cover type (pure: 6.5%, total: 10.6%; Table 2), carbon content (pure: 5.7%, total: 6.3%), and elevation (pure: 4.3%, total: 8.0%) followed by soil texture (pure: 3.9%, total: 7.1%) and human disturbance (pure: 3.8%, total: 7.0%; Table 2). A similar pattern was observed for fungal communities. Soil texture, elevation, human disturbance, and cover type were the most important factors explaining 3.5% (pure: 2.0%), 4.9% (pure: 2.1%), 6.2% (pure: 2.2%), and 3.8% (pure: 4.3%) of the variation in fungal communities, respectively (Table 2). Electrical conductivity and pH were not selected in the best fitting model, presumably because they did not substantially contribute to explaining community composition within the observed range, or because they were redundant (collinear) with parameters already included in the model.

**Table 2:** Effects of contextual parameters on variation in bacterial and fungal communities of studied soils.

Explanatory factors	Bacteria					Fungi			
	Effect	R²	F	df	p	R²	F	df	p
Soil texture	Pure	3.9	2.1	2, 37	0.001***	2.0	1.5	2, 36	0.004**
	Total	7.1	2.7	2, 42	0.001***	3.5	1.8	2, 41	0.001***
Cover type	Pure	6.5	2.8	2, 37	0.001***	4.3	2.0	2, 36	0.001**
	Total	10.6	3.6	2, 42	0.001***	3.8	1.9	2, 41	0.001***
Carbon content	Pure	5.7	4.1	1, 37	0.001***	3.2	2.5	1, 36	0.001***
	Total	6.3	3.9	1, 43	0.001***	3.5	2.6	1, 42	0.001***
Elevation	Pure	4.3	3.4	1, 37	0.001***	2.1	2.0	1, 36	0.001***
	Total	8.0	4.8	1, 43	0.001***	4.9	3.2	1, 42	0.001***
Human disturbance	Pure	3.8	3.1	1, 37	0.001***	2.2	2.0	1, 36	0.002**
	Total	7.0	4.3	1, 43	0.001***	6.2	3.8	1, 42	0.001***
Nitrogen content	Total	5.1	3.4	1, 43	0.001***	3.9	2.7	1, 42	0.001***
Conductivity	Total	1.3	1.6	1, 43	0.035*	0.1	1.1	1, 42	ns
pH	Total	1.3	1.6	1, 43	0.027*	0.6	1.2	1, 42	ns
All	Pure	31.5	3.9	7, 37	0.001***	19.1	2.5	7, 36	0.001***
	Total	32.1	3.1	10, 34	0.001***	19.5	2.0	10, 33	0.001***

Total and the pure effects (i.e. when controlling for all other factors of the analysis) of explanatory factors were calculated by using canonical redundancy analysis (RDA) models. The proportion of explained community variation is expressed as R^2^ values. Significances of the respective F-ratios were tested by performing 1000 Monte Carlo permutation tests and are indicated by *significant (P ≤ 0.05), **very significant (P ≤ 0.01), ***highly significant (P ≤ 0.001), and ns when not significant (P > 0.05). df: degrees of freedom (numerator, denominator).

We were not always able to isolate the effect of a particular parameter on bacterial community composition, while accounting for all of the other parameters in the model as evidenced by the differences between pure and total effects (Table 2). For instance, since samples with the highest electrical conductivity were all sandy soils collected at Shana from cyanobacteria-dominated crusts/mats, it was not possible to reliably attribute the differences observed in the bacterial communities at Shana to any of these parameters. Besides, bare soils were mostly collected at disturbed non-elevated locations, whereas lichen-crusts were mainly found at elevated undisturbed sites, resulting in a non-orthogonal sampling design and making a distinction between the effects of the different factors very difficult. Therefore, the pure effects of the parameters included in the RDA were considerably lower than the total effects (Table 2).

### Alpha and beta diversity analysis

**Figure 3:**
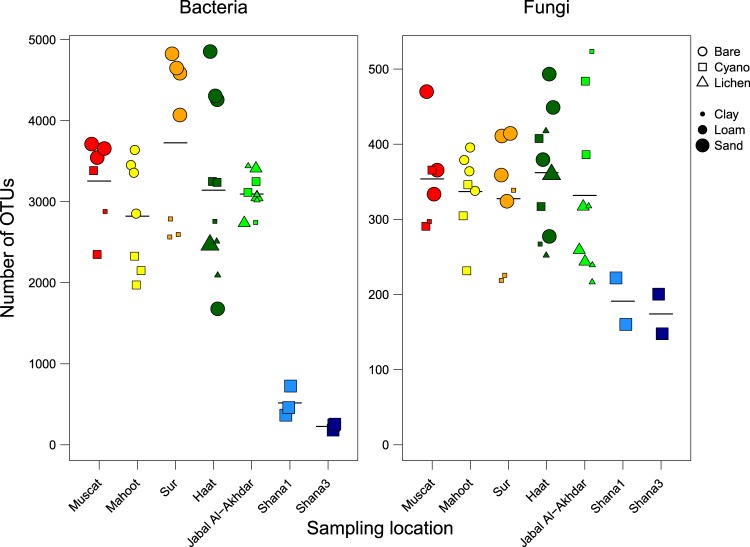
Bacterial and fungal richness (number of OTUs). Color, shape, and size of points indicate sampling location, surface cover type, and soil texture, respectively. The sample color codes are as in Fig. 2.

Bacterial and fungal diversity estimators were lower in the samples from Shana compared to all other samples (Fig. 3; Supplementary Table S1). For instance, bacterial and fungal species richness, as determined by number of OTUs, reached an average of 3196 ± 796 and 343 ± 80 in Muscat, Mahoot, Sur, Jabal Al-Akhdar and Haat soils but only 371 ± 200 and 183 ± 35 in Shana soils, respectively. Among the locations, where both bare and crusted soils were sampled, the average numbers of OTUs and Shannon index in bare soils at Mahoot, Sur and Haat were considerably higher than in crusted soils at these sites, whereas at Muscat similarly high numbers were reached by cyanobacteria-dominated crust (Fig. 3, Supplementary Table S1). The averages of OTU richness of crusted soils from all sites (except from Shana) were comparable.

**Figure 4:**
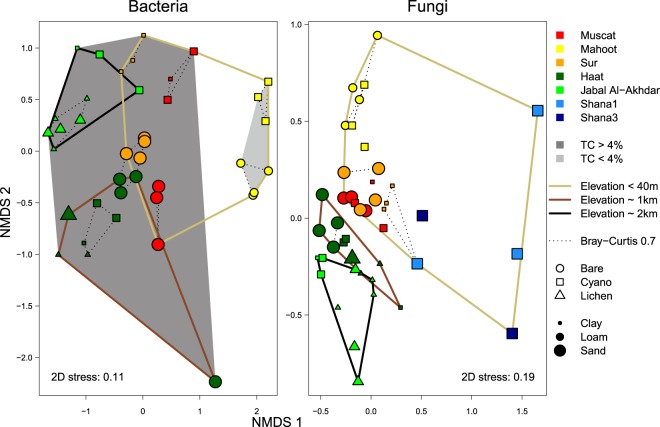
Non-metric multidimensional scaling (NMDS) plot based on Bray-Curtis dissimilarity of bacterial and fungal communities. Color, shape, and size of points indicate sampling location, surface cover type, and soil texture, respectively. Dotted lines show clusters with a maximum intra-group dissimilarity of 0.7. Solid lines group samples with similar elevation. Shaded hulls indicate organic carbon content (TC in %).

**Figure 5:**
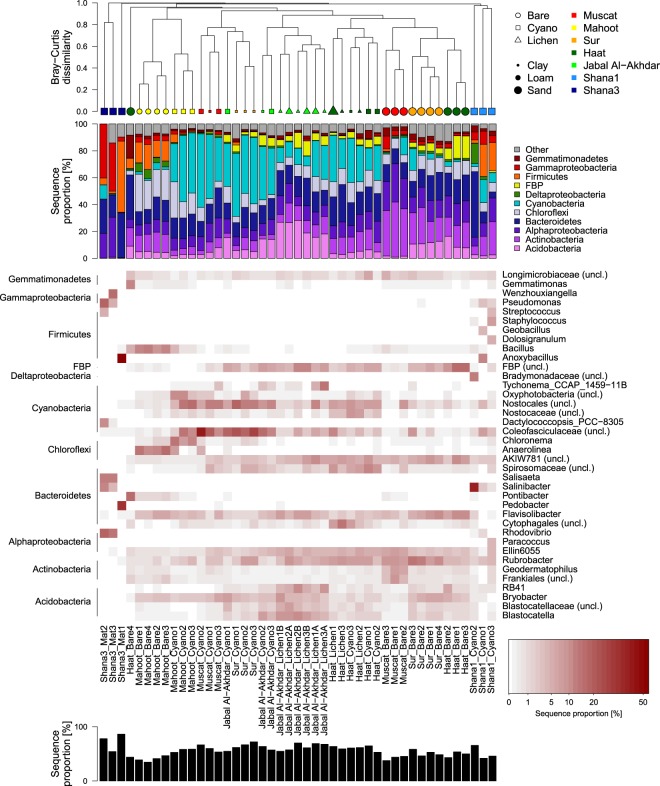
Bacterial community composition on phylum and genus level. Order of plots from top to bottom: hierarchical cluster diagram based on Bray-Curtis dissimilarity, bar plot of relative sequence proportions of dominant bacterial phyla (class-level taxonomy for Proteobacteria), heatmap of dominant genera (white: no sequences), total sequence proportion of genera displayed in heatmap.

**Figure 6:**
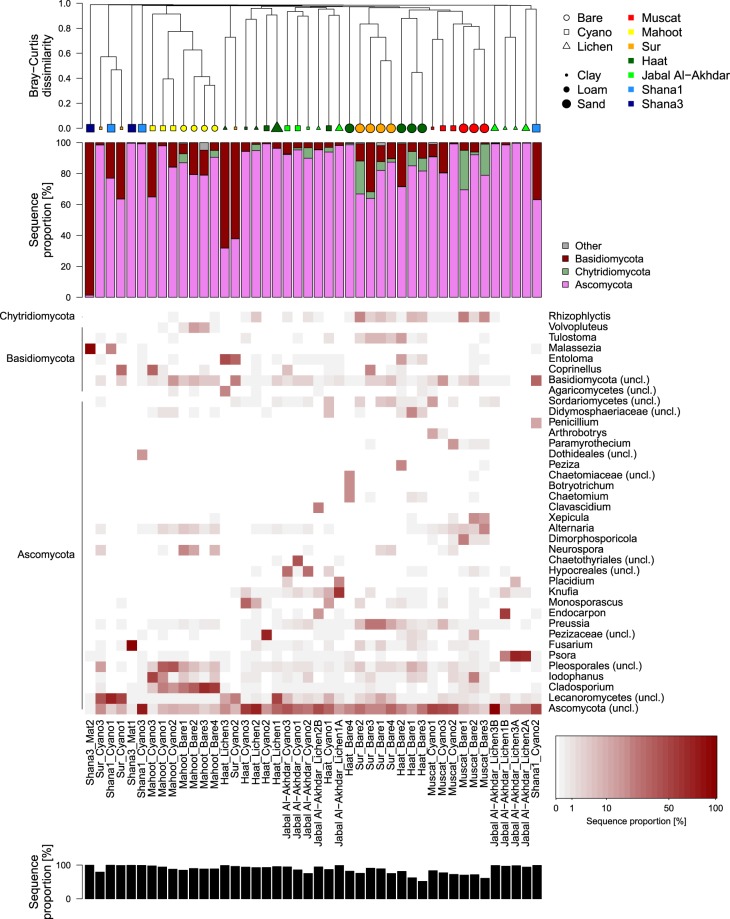
Fungal community composition on phylum and genus level. Order of plots from top to bottom: hierarchical cluster diagram based on Bray-Curtis dissimilarity, bar plot of relative sequence proportions of dominant bacterial phyla, heatmap of dominant genera (white: no sequences), total sequence proportion of genera displayed in heatmap.

When variations in bacterial and fungal communities among bare soils and biocrusts were visualized in a two-dimensional space using multidimentional scaling (NMDS), both communities showed distinct clusters according to the significant parameters determined by the RDA analysis (Fig. 4). Soils of the same cover type and from the same site clustered together, although there was a clear heterogeneity among replicates (Fig. 4). Bacterial communities of soils containing more than 4% carbon content were clearly different from those found in less than 4% carbon-containing soils from similar elevation and disturbance conditions (Fig. 4). The NMDS ordination further suggested a clustering of bacterial as well as fungal communities based on the elevation of the sampling site, which was, however, only moderately supported by ANOSIM (R = 0.31–0.57, P* < *0.05), due to a high intra-group dissimilarity. Bacterial communities of Shana samples, exposed to marine influence and characterized by high electrical conductivity, were very different from the other locations and formed a well-separated, although very heterogeneous, cluster (Fig. 5, ANOSIM R = 0.61–0.89, P < 0.002). Fungal communities at Shana were also distinct from the other sites (Fig. 4, ANOSIM R = 0.57–0.81, P < 0.002), although given the generally much higher heterogeneity of fungal communities (Fig. 6) this separation was not as pronounced as for the bacterial communities (Fig. 5).

Cluster analysis based on Bray-Curtis dissimilarities provided more details about the pattern in community dissimilarity than NMDS. It confirmed that bacterial and fungal communities clustered together based on cover type (i.e. bare, cyanobacteria- or lichen-dominated crust), as well as their sampling site (Figs 5 and 6). Bare soils were found in two clusters: the first cluster comprising Muscat, Sur and Haat samples, while the second cluster was formed by bare soil samples from Mahoot (Figs 5 and 6). In general, bacterial and fungal communities displayed a surprising congruency in diversity patterns.

### Variation in bacterial communities

In all bare and crusted soils, the majority of sequences belonged to Actinobacteria (average 13% across all samples), Alphaproteobacteria (12%), Bacteroidetes (16%) and Cyanobacteria (21%), encompassing between 32 to 83% of the total number of sequences per sample (Fig. 5). Notably a considerable proportion of all sequences (45%) could not be confidently classified on genus level. Actinobacteria exhibited their highest relative abundance in bare soils (21 ± 9% of total sequences), followed by lichen-dominated (11 ± 3%) and cyanobacteria-dominated crusts (7 ± 6%). In bare soils, the relative abundance of Actinobacteria was highest in Muscat soils, but lowest in Mahoot soils, whereas in cyanobacteria-dominated crusts, they were higher in Haat than in Jabal Al-Akhdar samples (Fig. 5). The relative abundance of Actinobacteria in Haat soils was comparable, regardless of the surface cover type, with an average of 16 ± 2% in the bare soil, 11 ± 4% in the cyanobacteria- and 13 ± 2% in the lichen-dominated crusts. In contrast, their relative abundance in lichen-dominated crusts from Jabal Al-Akhdar was considerably higher than in the cyanobacteria-dominated crusts (6–15% vs. 3–4% of total sequences, respectively). At Shana3, Actinobacteria were completely absent. At the genus level, most of the actinobacterial sequences across all samples belonged to *Rubrobacter*.

Among the proteobacterial classes, Alphaproteobacteria were most frequently encountered in all samples, with few sequences from Gammaproteobacteria (Fig. 5). The relative abundance of Gammaproteobacteria did not exceed 10% in any of the soils, except in Shana3 mats, where they made up between 3–40% of the total number of sequences, respectively (Fig. 5). Gammaproteobacterial sequences belonged mainly to the genera *Wenzhouxiangella*, *Pseudomonas*, *Delftia* and the *Burkholderia-Caballeronia-Paraburkholderia* complex. The contribution of Alphaproteobacteria to the total number of sequences did not vary much among the different surface cover types, with an average proportion between 11 and 14% across all samples. The highest relative abundance of Alphaproteobacteria was detected in Muscat bare soils and Shana3 mats, with up to 28% and 30% of total sequences, respectively. While in Muscat soils these sequences belonged mainly to *Sphingomonas (Ellin6055)*, they belonged to *Rhodovibrio* in Shana3 mats (Fig. 5). In Muscat soils, the relative abundance of Alphaproteobacteria in bare soils was comparatively higher than in cyanobacteria-dominated crusts (24 ± 4% vs. 13 ± 2%).

Bacteroidetes constituted between 5 to 49% of the total number of sequences in each sample (average 17 ± 7%). Both of these extremes were recorded in samples from Shana1 (Fig. 5). Samples from Shana3 and Haat generally displayed higher proportions of Bacteroidetes (16–33%) compared to the remaining locations with similar proportions between 10 and 22%. At each sampling location, the relative abundance of Bacteroidetes among bare and crusted soils was comparable (Fig. 5). In Shana soils, most Bacteroidetes sequences belonged to *Salisaeta*, *Salinibacter* and *Pedobacter*, although these genera were barely detectable in any other soil (<0.2% of total sequences; Fig. 5). Conversely, sequences affiliated to *Flavisolibacter* were found in all bare and crusted soils from the other sites (Fig. 5).

Cyanobacteria exhibited the most remarkable differences between the different types of soils and biocrusts in all locations (Fig. 5). While cyanobacteria were barely detectable in most bare soil samples (average of 4 ± 5%), their average percentage across the cyanobacteria- and lichen-dominated crusts reached 29 ± 18% and 20 ± 9%, respectively (Fig. 5). In bare soils, cyanobacteria exhibited their highest relative abundance in one of the Muscat and Sur samples (13% and 16% of total sequences, respectively) but made up only less than 5% in each of the other samples (Fig. 5). The relative abundance of cyanobacteria in cyanobacteria-dominated crusts was notably lower at Shana1 compared to all other sites and they were almost absent in the Shana3 mats, with the exception of *Dactylococcopsis*, which constituted 10% of the sequences in one sample (Fig. 5). At Jabal Al-Akhdar, cyanobacteria had higher relative abundance in cyanobacteria- than in lichen-dominated crusts with an average of 35 ± 4 and 16 ± 8%, respectively, but this difference was not observed in samples from Haat (25 ± 8 vs. 28 ± 6%). Notably, the most frequent cyanobacterial OTUs could not be classified on genus level, but were affiliated with uncultured representatives of the Nostocales. Closest relatives were *Coleofasciculus* (sequence identity 89–95%), *Loriellopsis* (88–89%), *Planktothricoides* (89%), *Brasilonema* (92–96%, Supplementary Dataset 1). These so far not well characterized OTUs dominated the cyanobacterial communities of most crusts, except those from Shana, but displayed variable distribution among different samples. For instance, while most presumptive *Coleofasciculus* OTUs were prevalent in cyanobacteria-dominated crusts of Muscat and Sur samples, other presumptive *Coleofasciculus* as well as potential *Brasilonema* OTUs were frequently found in cyanobacteria- and lichen-dominated crusts of Haat, and an OTU most closely related to *Loriellopsis* characterized the cyanobacteria-dominated crusts of Mahoot.

Sequences belonging to Acidobacteria were encountered in all soils at a relative abundance of less than 8% of the total number of sequences in each sample, except in bare soils of Sur and Haat (8–18% of total sequences in each sample) and crusted soils of Jabal Al-Akhdar (14–15% in the cyanobacteria-dominated crusts and 16–28% in lichen-dominated crusts). Most of these sequences belonged to the genus *Bryobacter* and representatives of the Blastocatellaceae (Fig. 5). While of low overall sequence abundance, Firmicutes contributed substantially to samples from bare soils of Mahoot (12–18%), and cyanobacteria-dominated crusts of Shana1 (2–24%) and Shana3 (2–53%). Similarly, Chloroflexi were only found in high proportions in both bare soils and cyanobacteria-dominated crusts of Mahoot (12–28%; Fig. 5).

### Variation in fungal communities

Fungal sequences belonged to three main phyla (i.e. Ascomycota, Chytridiomycota, and Basidiomycota), with an average of 83 ± 20% of the total number of sequences belonging to Ascomycota across all samples (Fig. 6). Similar to the bacterial dataset, a large proportion of fungal sequences (49%) remained unclassified on genus level. Ascomycota were predominantly encountered in most bare and crusted soils (≥50% of total sequences), except three samples from Shana3, Haat, and Sur (Fig. 6). This group constituted 99% of the total sequences in one microbial mat from Shana3 (Shana3_Mat1) but was almost absent in another mat sample (Shana3_Mat2; Fig. 6). This high heterogeneity between sampling locations and cover type, but also between replicate samples from similar conditions, was even more pronounced at higher taxonomic resolution, where each sample was often dominated by a (different) single genus. Patterns of Ascomycota genera among samples were further obscured by a large proportion of Ascomycota OTUs, which all had less than 85% sequence identity to known fungal species (Supplementary Dataset 2), but which showed highly variable sequence proportions among samples. For instance, unclassified OTUs of the Lecanoromycetes characterized cyanobacterial-dominated crusts in Sur and Shana1. Among classified representatives of the Ascomycota, *Cladosporium* was found predominantly in both bare soils (28–66%) and cyanobacteria-dominated crusts (4–23%) of Mahoot, while *Neurospora* was mostly restricted to bare soils from this location (Fig. 6). OTUs of the genera *Psora*, *Endocarpon*, *Knufia*, and *Placidium* dominated most of the lichen-crusts of Jabal Al-Akhdar comprising up to 69%, 58%, 60%, and 21% of the fungal community, respectively (Fig. 6). The highest proportions of *Fusarium* and *Penicillium* were encountered at Shana3 and Shana1, respectively, however, only in one out of three replicate samples. The genera *Dimorphosporicola*, *Xepicula*, and *Alternaria* were predominantly found in bare soils from Muscat with up to 24%, 17%, and 21%, respectively, whereas they comprised mostly less than 1% in all other sample (Fig. 6). In bare soils of Sur, *Preussia* was consistently enriched (5–27%), while *Monosporascus* was found in high proportions in several crusts from Haat (Fig. 6).

The relative abundance of Chytridiomycota was considerably higher in bare soils with up to 26% compared to cyanobacteria- and lichen-dominated crusts, where they accounted for less than 7% and 4% of the total number of sequences in each sample, respectively (Fig. 6). Nevertheless, there were clear differences among replicates from the same site (Fig. 6), with percentages ranging between 2–26%, 0.3–9%, and 2–21% in bare soils from Muscat, Haat and Sur. Most Chytridiomycota sequences belonged to the genus *Rhizophlyctis*, which in some replicates accounted for 8–25% of the sequences in bare soils from Muscat, Sur and Haat sites (Fig. 6).

Sequences belonging to Basidiomycota were also detected in bare soils, mat samples, cyanobacteria- and lichen-dominated crusts from all sites (Fig. 6). Their relative abundance in a mat sample from Shana3 and a cyanobacteria- and a lichen-dominated crusts from Sur and Haat was highest and reached 62 to 99% of the total number of sequences (Fig. 6). The main genera determined for this phylum were *Malassezia*, *Coprinellus*, *Volvopluteus*, *Entoloma* and *Tulstoma*, of which *Malassezia* was found mainly in Shana samples, while *Volvopluteus* was characteristic for bare soils from Mahoot, and *Tulstoma* for bare soils from Sur (Fig. 6). Sequences belonging to *Coprinellus* accounted for 33% of the total number of sequences in one cyanobacteria-dominated crust from Mahoot and Sur (Fig. 6).

## Discussion

Field observations have shown that lichen-dominated biocrusts become more prevalent as aridity decreases and precipitation rates increase^20,23^. This is congruent with our observations at the elevated sites of Haat and Jabal Al Akhdar and with the previously reported lichen vegetation of the Arabian Peninsula, Colorado Plateau and Gurbantunggut Desert^46,47^. At the Jabal Al Akhdar site, the higher annual precipitation and the mountain slopes supported greater cover of lichens and the samples were characterized by fine-textured soils.

While the relative abundance of Cyanobacteria was much higher in crusted than in bare soils, the relative abundance of Actinobacteria showed an opposite trend. Shifts in microbial communities at different successional stages of crust development have been previously reported^18,48–50^. This is ascribed to the fact that growth of Cyanobacteria results in higher levels of organic matter and nutrients in crusted soils, relative to bare soils, thus promoting the growth of different microbial communities^51,52^. The improvement in soil physiochemical properties under crusts also promotes hyphal proliferation and fungal growth^53,54^. However, the competition for organics and nutrients in crusts could result in the growth of highly specific and competitive microbial populations and this could explain our lower OTU richness of bacteria and fungi in crusted than in bare soils.

The detected bacterial phyla including Cyanobacteria, Actinobacteria and Proteobacteria, were also numerically abundant in other crusts from the Colorado Plateau and Sonoran Desert^55,56^. The Cyanobacteria *Coleofasciculus* (formerly *Microcoleus*), *Brasilonema* (a genus placed within *Scytonema* in the silva reference database^41^ and Nostocales have been previously encountered in crusts from Oman and elsewhere^30,31,56–58^. The prevalence of *Coleofasciculus* spp. in most crusts from all locations is consistent with previous observations, and highlights their crucial role in the consolidation of soil particles and crust formation^55,56,59^. Actinobacteria, exhibiting their highest relative abundance in bare soils, are common in soil environments and play a vital role in the decomposition of organic matter and recycling of nutrients^60^. Actinobacteria was the most dominant phylum in biocrusts from the Colorado Plateau, Sonoran Desert and Tengger Desert, China^17,48,55,56^, but were detected at a very low dominance in biocrusts from the Gurbantunggut Desert and Cape Code Seashore^18,61,62^. Bacteroidetes and Proteobacteria, which are known to produce exopolysaccharides, may contribute to soil stabilization and biocrust formation^56^. Proteobacteria and Bacteroidetes were shown to increase with biocrust formation in the Gurbantunggut Desert^18^. Also in biocrusts of the Succulent Karoo in South Africa, the relative abundance of some bacterial phyla (i.e., Acidobacteria, Chloroflexi, Verrucomicrobia) was higher, whereas that of Thermi was lower compared to uncrusted soils^50^. The detection of some similar taxa in bare soils and cyanobacteria- and lichen-dominated crusts suggests that some bacterial populations are retained during the crust development process.

The dominance of Ascomycota in our biocrusts has also been shown for biocrusts from the Negev Desert, Wyoming and Utah, Colorado Plateau, and the Chihuahuan Desert^63–67^. While Ascomycota showed a similar relative abundance across all bare and crusted soils, Chytridiomycota were more prevalent in bare than in crusted soils. Members of Chytridiomycota have not been detected in any biocrusts studied up to date, except at a low abundance in the Oman and the Chihuahuan deserts^65,68^. This suggests that Chytridiomycota belong to an early stage of crust development, indicating their tolerance to stress environments. The dominance of different fungi in bare soils compared with biocrusts indicates successional changes of fungal communities during crust formation from bare to lichen-dominated crusts, which have been previously demonstrated^69^. The dominance of a single fungus in every soil supports the notion that fungal succession occurs through the selection of the best-adapted species to a certain ecological niche^69,70^. The fungi belonging to the detected genera in our biocrusts are often very common components of crusts and aridland soils worldwide^63,64,71^. Interestingly, the lichen-forming *Endocarpon* was described for the first time for crusts from this region, whereas *Psora* has been known to be widespread^68,72^.

### Crust microorganisms and soil characteristics

Microbial communities in our crusts were strongly affected by soil texture and carbon (and nitrogen) contents as could be inferred from RDA analysis. This is expected since clear differences in soil texture were observed, ranging from fine loamy texture only in Mahoot samples to coarse sandy texture in most bare soils and Shana crusts and mats. Soil texture affects crust development by influencing soil stability, aeration and water retention ability, as coarser soils are unstable and lose more water relative to fine soils^73^. Previously, it was shown that cyanobacteria develop on sandy soils whereas lichens preferably grow on fine texture soils^74^. However, biocrusts have also been shown to influence soil texture, as soil mean particle size decreased with succession of biocrusts of the Gurbantunggut Desert of northwestern China^75^. Lichens were also found associated with high soil N and C content^76^. This is in agreement with the greater cover of lichens at the elevated Jabal Al-Akhdar and Haat sites, characterized by high levels of carbon and nitrogen and a fine soil texture (Table 1, Fig. 2). Also for N and C contents, it is difficult to distinguish between the preference and impact of biocrust types on these factors, as well-developed biocrusts have been shown to also contribute to increased N and C contents in soils^77,78^.

Among the bacterial genera encountered in this study, the relative abundance of presumptive *Coleofasciculus*, and *Blastocatella*, was high in biocrusts with loam and clay textures in Jabal Al Akhdar, Haat, Sur and Muscat sites, whereas *Bacillus* exhibited their maximum relative abundance in biocrusts with loamy texture at the Mahoot site. The bacterial phyla Chloroflexi and Firmicutes, detected in Mahoot soils, are known to include species that are either strictly or facultatively anaerobic^79,80^. It is plausible that after heavy rainfalls, the fine-textured soil in Mahoot may cause limited oxygen diffusion, thus resulting in the development of anaerobic niches. Sequences belonging to Firmicutes and Chloroflexi have been previously reported from different bare desert soils and crusts from Oman, Atacama, Gurbantunggut, Tabernas, South African and Sonoran deserts^18,50,80–84^. Similar to bacterial communities, the distribution of fugal genera was also driven by soil texture. For instance, the fungal genera *Cladosporium* and *Neospora* were associated with loamy texture at Mahoot, the lichen-forming *Psora*, *Endocarpon* and *Monosporascus* were detected in loamy and clayey soils of Jabal Al-Akhdar and Haat, and *Rhizophlyctis* and *Malassezia* were mainly detected in coarse soils of Muscat and Shana, respectively.

### Crustal microorganisms and elevation

RDA analysis indicated elevation to be a major contributor to variations in crust bacterial and fungal communities, and this was further supported by NMDS plots. Sites at different elevations clearly vary in climate, precipitation amounts and patterns, temperature regimes (daily and seasonal), UV and solar radiation, moisture and soil texture and chemistry. All these parameters have been shown to directly affect crustal microbial communities^10,15,64^. For instance, microbial communities in crusts from the Negev and Mojave Deserts have been shown to vary depending on altitude and annual precipitation^64^. Crust abundance and development was shown to increase with increasing moisture and precipitation levels, whereas with increasing aridity, biocrusts were restricted to cyanobacteria-dominated types^10,85^. Presumptive *Brasilonema*, *Blastocatella*, and some Nostocales OTUs showed the strongest differences along the elevation gradient. The sequence proportion of these bacterial genera increased with elevation, with the exception of Nostocales OTUs, which exhibited divergent trends. Cyanobacteria could play an active role in nitrogen fixation, contributing to the measured higher levels of N at the elevated compared to the non-elevated sites (Fig. 2). Indeed, previous research has demonstrated an increase in nitrogen fixation rates with increasing temperature, precipitation, and altitude^86^.

While the relative abundance of the lichen genera *Psora* and *Endocarpon* increased with elevation, showing their maximum percentage at Jabal Al- Akhdar, the relative abundance of *Cladosporium* and several unclassified Ascomycota decreased. These genera accounted for most of the differences in fungal communities between elevated and non-elevated biocrusts. The gelatinous fungal material surrounded by *Endocarpon* can absorb and retain water during precipitation events^87^. Therefore, elevation could promote the growth of fungi that are adapted to sporadic precipitation events.

### Marine influence on crustal and mat microorganisms

The remarkable difference, especially in bacterial, but also in fungal communities, of the Shana samples, as shown by cluster and NMDS analyses, suggests that salinity may be a major determinant shaping these biocrust communities. This marine influence lead to significantly higher electrical conductivities and lower nutrient concentrations in Shana crusts (Table 1) and shifted the microbial communities. The infrequent exposure to seawater during high tides, followed by desiccation due to very high evaporation rates, resulted in salt precipitation in the Shana1 crusts. The Shana3 mat samples were regularly inundated, mostly soft, and had a layered structure with greenish and pink strata. Thus, the samples of Shana1 and Shana3 reflect a transition from salt-influenced biocrusts to intertidal mats. While the Shana1 crusts comprised Cyanobacteria, Actinobacteria, and Acidobacteria, these phyla were almost completely absent in Shana3 mats, where in some samples *Rhodovibrio*, *Pedobacter* and *Anoxybacillus* occurred in large numbers. This transition from microbial mats to crusts in coastal deserts has been previously described for the Sebkhas of Tunisia^15^. Earlier studies on various ecosystems demonstrated a decrease in microbial diversity with increasing salinity^88–91^. This is consistent with our observation, where OTU richness of both bacterial and fungal communities in Shana samples was indeed significantly lower than in other crusts. At Shana1, cyanobacteria were made up by halotolerant types such as *Dactylococcopsis* and presumptive *Coleofasciculus*, which are typically found in hypersaline mats^92,93^. The increased proportion of Bacteroidetes, known for their ability to degrade polymeric substances, in some samples could be linked to the increased production of EPS by cyanobacteria under salt stress^94,95^. The detection of many halophilic genera such as *Salisaeta* and *Salinibacter* is also consistent with the high salinities in these samples.

Elevated soil salinities are known to be less favourable for the development of lichen-dominated crusts^19^. Although high salinities do not support the growth of fungi^96^, several types of fungi were detected in Shana samples. The most prevalent fungi in Shana biocrusts were *Malassezia*, *Fusarium*, *Penicillium*, as well as many so far unclassified OTUs. Fungi belonging to some of these genera have been reported for other crusts from Oman, Colorado Plateau, Gurbantunggut Desert, Utah and Wyoming^63,67–69^. Nevertheless, their detection predominantly in Shana biocrusts suggests the ability of these genera to tolerate elevated salinities. Indeed, various taxa of *Penicillium* and *Fusarium* have been detected in salterns and hypersaline microbial mats and played a role in the degradation of EPS and growth on cyanobacterial exudates^97,98^.

We conclude that spatial heterogeneity in bacterial and fungal communities in the cyanobacteria- and lichen-dominated crusts from the central and coastal deserts of Oman is highly correlated with soil and site characteristics. Striking shifts in microbial communities were detected in crusts along an elevation gradient and in crusts/mats exposed to marine influence. Our results confirm previously reported effects of the studied parameters on other crusts. This strengthens the notion that crust microbial communities are highly selective to fit specific habitat conditions. Furthermore, the large proportion of unclassified OTUs in our data set emphasizes that biological soil crusts in Oman are a severely understudied environment.

## Supplementary information

Supplementary Table S1

Supplementary Dataset1

Supplementary Dataset2
