# Supplementary material for: Habitat-dependent composition of bacterial and fungal communities in biological soil crusts from Oman
Source: Sci Rep. 2019 Apr 23;9:6468. doi: 10.1038/s41598-019-42911-6 (PMC6478931; doi:10.1038/s41598-019-42911-6)
Supplement: Supplementary file 1 — Supplementary Table S1 [file 41598_2019_42911_MOESM1_ESM.pdf]

Habitat-dependent composition of bacterial and fungal communities in biological soil crusts from Oman

Raeid M. M. Abed[a*], Alexandra Tamm[b], Christiane Hassenrück[c], Ahmed Al-Rawahi[a], Emilio Rodríguez-Caballero[b,d], Sabine Fiedler[e], Stefanie Maier[b], Bettina Weber[b]

[a] Sultan Qaboos University, College of Science, Biology Department, P.O. Box: 36, postal code 123, Al Khoud, Sultanate of Oman

[b] Multiphase Chemistry Department, Max Planck Institute for Chemistry, Hahn-Meitner-Weg 1, D-55128 Mainz, Germany

[c] Tropical Marine Microbiology, Department of Biogeochemistry and Geology, Leibniz Centre for Tropical Marine Research, Bremen, Germany

[d] Departamento de Agronomia, Universidad de Almeria, Almería, Spain

[e] Johannes Gutenberg-University, Institute for Geography, Mainz, Germany

[*]Corresponding author: Raeid M. M. Abed

Mailing address: Sultan Qaboos University, College of Science, Biology Department, P.O. Box 36, Al Khoud, postal code 123, Muscat, Sultanate of Oman. Tel: +968-24142406 e-mail: rabed@mpi-bremen.de

Supplementary Table S1. Diversity estimators including number of OTUs (nOTUs), Shannon index (Shannon) and inverse Simpson index (invS) of studied soils from different locations in the coastal and central deserts of the Sultanate of Oman

Supplementary Dataset1. Blast results for the most abundant OTUs of each of the bacterial genera displayed in main figure 5. Sequences were blasted against the 16S bacterial and archaeal sequence collection of NCBI, using megablast (date accessed: 03.03.2019). Full names and taxonomic paths of the 10 best hits (if applicable) were retrieved using the R package rentrez [100]. The genus classification obtained with the RDP Naïve Bayesian classifier implemented in dada2 is also provided.

Supplementary Dataset2. Blast results for the most abundant OTUs of each of the fungal genera displayed in main figure 5. Sequences were blasted against the full nucleotide database of NCBI, using megablast (date accessed: 03.03.2019). Full names and taxonomic paths of the 10 best hits (if applicable) were retrieved using the R package rentrez [100]. The genus classification obtained with the RDP Naïve Bayesian classifier implemented in dada2 is also provided.

Table S1. Diversity estimators including number of OTUs (nOTUs), Shannon index (Shannon) and inverse Simpson index (invS) of bare and crusted soils from different locations in the coastal and central desert of the Sultanate of Oman

Sampling site	Type/ Replicate	Bacteria			Fungi		
		nOTUs	Shannon	invS	nOTUs	Shannon	invS
Muscat	Bare1	3714	7.10	370.89	334	3.33	9.70
	Bare2	3546	6.96	208.90	470	3.55	13.82
	Bare3	3658	7.36	542.31	366	3.04	10.67
	Cyano1	2879	6.04	80.15	297	3.00	7.99
	Cyano2	2349	5.05	14.12	396	3.06	6.03
	Cyano3	3384	6.70	188.22	219	2.50	5.43
Sur	Bare1	4588	7.40	334.39	411	3.73	13.76
	Bare2	4828	7.61	403.69	359	3.30	13.51
	Bare3	4072	6.93	105.39	324	3.14	9.34
	Bare4	4652	7.57	437.40	414	3.89	23.30
	Cyano1	2564	5.53	38.33	226	1.65	3.07
	Cyano2	2788	6.15	106.19	219	1.89	4.11
	Cyano3	2596	5.96	86.38	339	2.58	4.93
Mahoot	Bare1	3454	6.99	293.48	364	3.23	10.08
	Bare2	3640	7.08	340.76	396	3.30	10.42
	Bare3	2854	6.69	240.83	338	2.85	5.35
	Bare4	3359	7.02	318.74	379	3.11	7.01
	Cyano1	2328	6.30	179.97	305	2.64	6.56
	Cyano2	2149	5.62	60.76	346	3.15	8.63
	Cyano3	1972	5.43	45.92	232	1.77	3.09
Haat	Bare1	4261	7.37	466.13	493	4.13	22.73
	Bare2	4855	7.71	856.67	380	3.25	12.14
	Bare3	4308	7.37	454.70	449	4.10	30.10
	Bare4	1680	4.95	25.31	277	2.44	7.25
	Cyano1	3250	6.46	78.25	408	3.32	11.33
	Cyano2	3239	6.59	190.90	317	1.61	1.84
	Cyano3	2758	6.13	90.12	267	2.09	4.09
	Lichen1	2466	5.98	118.51	360	2.23	3.36
	Lichen2	2511	6.05	89.31	418	2.09	2.30
	Lichen3	2092	5.61	51.61	252	2.20	5.87
Jabal Al-Akhdar	Cyano1	3114	5.97	71.11	484	2.76	4.27
	Cyano2	2743	6.29	139.93	523	3.69	10.36
	Cyano3	3248	6.66	214.28	386	2.50	4.88
	Lichen1A	3056	6.58	152.00	317	1.81	2.74
	Lichen2A	3410	6.75	190.99	244	1.73	2.35
	Lichen3A	3020	6.25	41.75	216	1.19	1.96
	Lichen1B	3441	6.79	155.80	239	1.57	2.48
	Lichen2B	3088	6.59	171.20	318	2.30	5.69
	Lichen3B	2736	6.38	149.58	259	0.90	1.51

Shana1	Cyano1	365	4.55	56.14	160	1.22	1.85
	Cyano2	725	4.96	39.33	222	1.79	3.56
	Cyano3	459	4.99	92.65	NA	NA	NA
Shana3	Mat1	242	1.68	3.41	200	0.49	1.15
	Mat2	181	3.00	15.81	148	0.71	1.30
	Mat3	254	2.96	12.63	NA	NA	NA
